# Video analysis in basic skills training: a way to expand the value and use of BlackBox training?

**DOI:** 10.1007/s00464-017-5641-7

**Published:** 2017-06-29

**Authors:** Ninos Oussi, Constantinos Loukas, Ann Kjellin, Vasileios Lahanas, Konstantinos Georgiou, Lars Henningsohn, Li Felländer-Tsai, Evangelos Georgiou, Lars Enochsson

**Affiliations:** 1 The Center for Advanced Medical Simulation and Training (CAMST), Karolinska University Hospital, Stockholm, Sweden; 2 Division of Surgery, Department of Clinical ScienceIntervention and Technology (CLINTEC), Karolinska Institutet, Stockholm, Sweden; 3 Center for Clinical Research Sörmland, Uppsala University, Uppsala, Sweden; 4 Medical Physics Lab-Simulation Center, Medical School, National and Kapodistrian University of Athens, Athens, Greece; 5 Division of Urology, Department of Clinical ScienceIntervention and Technology (CLINTEC), Karolinska Institutet, Stockholm, Sweden; 6 Division of Orthopedics and Biotechnology, Department of Clinical ScienceIntervention and Technology (CLINTEC), Karolinska Institutet, Stockholm, Sweden; 7 Division of Surgery, Department of Surgical and Perioperative Sciences, Umeå University, Umeå, Sweden; 8 Division of Surgery, Department of Surgical and Perioperative Sciences, Umeå University, 971 80 Luleå, Sweden

**Keywords:** BlackBox trainer, MIST-VR simulation, Virtual reality, Video analysis

## Abstract

**Background:**

Basic skills training in laparoscopic high-fidelity simulators (LHFS) improves laparoscopic skills. However, since LHFS are expensive, their availability is limited. The aim of this study was to assess whether automated video analysis of low-cost BlackBox laparoscopic training could provide an alternative to LHFS in basic skills training.

**Methods:**

Medical students volunteered to participate during their surgical semester at the Karolinska University Hospital. After written informed consent, they performed two laparoscopic tasks (PEG-transfer and precision-cutting) on a BlackBox trainer. All tasks were videotaped and sent to MPLSC for automated video analysis, generating two parameters (Pl and Prtcl_tot) that assess the total motion activity. The students then carried out final tests on the MIST-VR simulator. This study was a European collaboration among two simulation centers, located in Sweden and Greece, within the framework of ACS-AEI.

**Results:**

31 students (19 females and 12 males), mean age of 26.2 ± 0.8 years, participated in the study. However, since two of the students completed only one of the three MIST-VR tasks, they were excluded. The three MIST-VR scores showed significant positive correlations to both the Pl variable in the automated video analysis of the PEG-transfer (RSquare 0.48, *P* < 0.0001; 0.34, *P* = 0.0009; 0.45, *P* < 0.0001, respectively) as well as to the Prtcl_tot variable in that same exercise (RSquare 0.42, *P* = 0.0002; 0.29, *P* = 0.0024; 0.45, *P* < 0.0001). However, the correlations were exclusively shown in the group with less PC gaming experience as well as in the female group.

**Conclusions:**

Automated video analysis provides accurate results in line with those of the validated MIST-VR. We believe that a more frequent use of automated video analysis could provide an extended value to cost-efficient laparoscopic BlackBox training. However, since there are gender-specific as well as PC gaming experience differences, this should be taken in account regarding the value of automated video analysis.

Laparoscopic simulation training to proficiency levels has been shown to increase both basic skills [1–5] and the speed and precision of laparoscopic cholecystectomies [6–8]. However, the training necessary to achieve sufficient increased skills levels has usually been performed on laparoscopic high-fidelity simulators (LHFS) that all carry a substantial price tag [7, 9, 10]. Thus, the number of simulators necessary to satisfy the increasing needs for surgical training by medical students and residents is usually not met due to cost and their limited availability, obliging the trainees to overcome geographical barriers and travel to specialized simulation centers.

Basic skills training in BlackBoxes within a structured curriculum is a less costly alternative to LHFS-simulation training and has been shown to provide improvement of both basic as well as laparoscopic technical skills [11, 12]. However, it also has significant drawbacks in that this type of training does not provide feedback to the student or resident in regard to their performance [10, 13]. Thus, new methods of increasing the value of BlackBox training are warranted [10].

Various motion tracking systems have been proposed in the literature to address issues relevant to subjective and manual evaluation of surgical performance [14]. Other approaches employed multisensory modules for evaluating the quality of surgical maneuvers [15]. Hand motion analysis has also been combined with VR simulators for correlating technical errors with kinematic parameters [16], and for expertise classification [17]. A system that synchronizes motion tracking and video capture during laparoscopic cholecystectomy performance was proposed in the article by Dosis et al. [18]. The use of motion tracking for objective assessment of laparoscopic skills in the operating room was proposed by Aggarwal et al. [19]. The aforementioned works have highlighted the value of motion tracking for performance analysis and evaluation both in the simulation and clinical setting. Recently, some methods employed video analysis for the evaluation of laparoscopic skills, with encouraging results [20, 21].

There is some evidence of a positive relationship between video gaming experience and the acquisition of laparoscopic simulator surgical skills. However, the results are conflicting as there is a lack of a standardized scoring system and, therefore, further research on this subject is advised [22–24]. Glassman et al. in a recent systematic review concluded that there is a very limited amount of evidence to support that the use of video games enhances laparoscopic simulation performance [25]. Therefore, it would be of interest to study whether factors like PC gaming experience or gender differences could play a role in the efficacy and outcome of BlackBox training.

The aim of this study was to analyze if automated video analysis of basic skills training performance could be a valid method of increasing the value of BlackBox training and if it, in part, could provide an alternative to high-fidelity skills training in simulators, and furthermore to analyze if there were gender- or PC gaming experience-specific differences in the efficacy of BlackBox training. This study was a European collaboration within the framework of ACS-AEI.Query.

## Methods

### Participants and procedures

Thirty-one medical students (19 females and 12 males) with a mean age of 26.2 ± 0.8 (Mean ± SEM) were recruited for this study during their surgical semester at the Karolinska University Hospital. The study was conducted at the Center for Advanced Medical Simulation and Training (CAMST), Karolinska Institutet, Stockholm Sweden and the video analysis was carried out at MPLSC, Athens University Medical School, Athens, Greece.

**Fig. 1:**
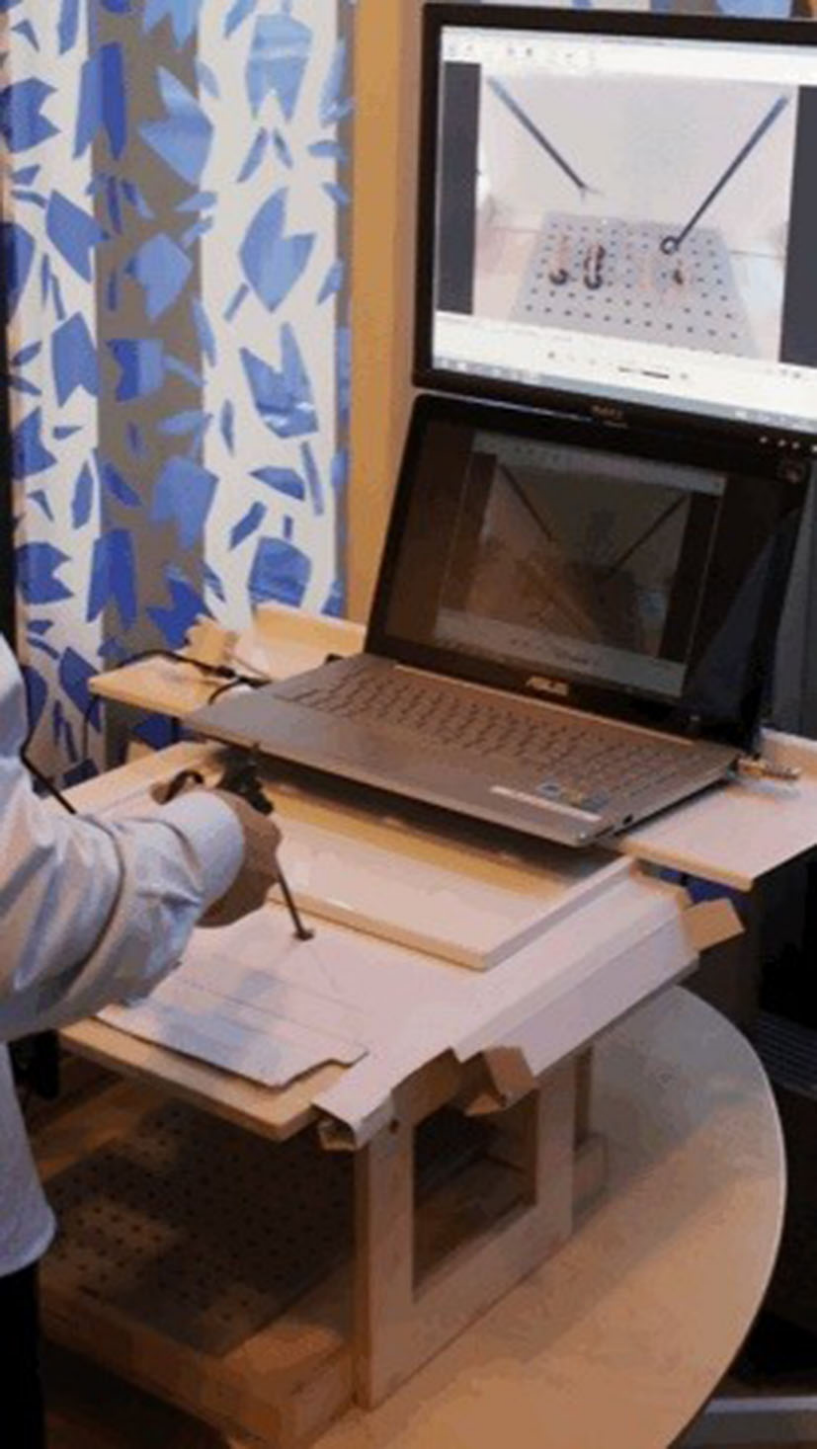
The BlackBox; a *box* consisting of wood-material, with three holes in the roof, one for a camera and two for the laparoscopic instruments

Students were recruited on a voluntary basis, when the study was presented to them, at the beginning of their surgical semester. After signing informed consent forms, the recruited students performed two laparoscopic tasks (PEG-transfer and cutting a circular gauze) on a BlackBox (built by and given to us by courtesy of MPLSC, Athens University, Greece) (Fig. 1). PEG-transfer and Precision-cutting were the tasks chosen on the reason that both are part of the Fundamentals of Laparoscopic Surgery (FLS) Technical Skills Proficiency-Based Training Curriculum.^1^ All the tasks were videotaped with a preinstalled web-camera and sent to MPLSC for automated video analysis. The students also performed three final tests on the validated Minimally Invasive Surgical Trainer (MIST-VR, Mentice, Gothenburg, Sweden) [26]. The data were gathered between September and December 2014 and the automated video analysis of the recorded procedures was carried out in 2015.

Oral instructions were given to each student before the study. None of the students had any prior surgical or laparoscopic experience, nor did they have any prior training before the task. However, some of the students had prior simulation training experience. The BlackBox is a wooden box with three holes located on the top, one for a camera and two for the laparoscopic instruments (Fig. 1). The camera used was a web-camera [Logitech, with a processor image resolution of 720 × 576 and a frame rate of 25 frames per second (fps)]. Inside and on the floor of the BlackBox, materials for conducting the different tasks were set up. A plastic frame consisting of wooden plugs (pegboard) and rubber rings was used for the PEG-transfer task, and for the Precision-cutting task, screws and nuts were used to hold a 10 × 10 cm gauze marked with two circles. For the above-mentioned tasks, laparoscopic graspers and scissors, marked with different colored markers on their distal end were used. The fixed web-camera was connected to a laptop for the recording of the tasks, and a flat monitor was used for the visualization of the tasks carried out (Fig. 1).

**Fig. 2:**
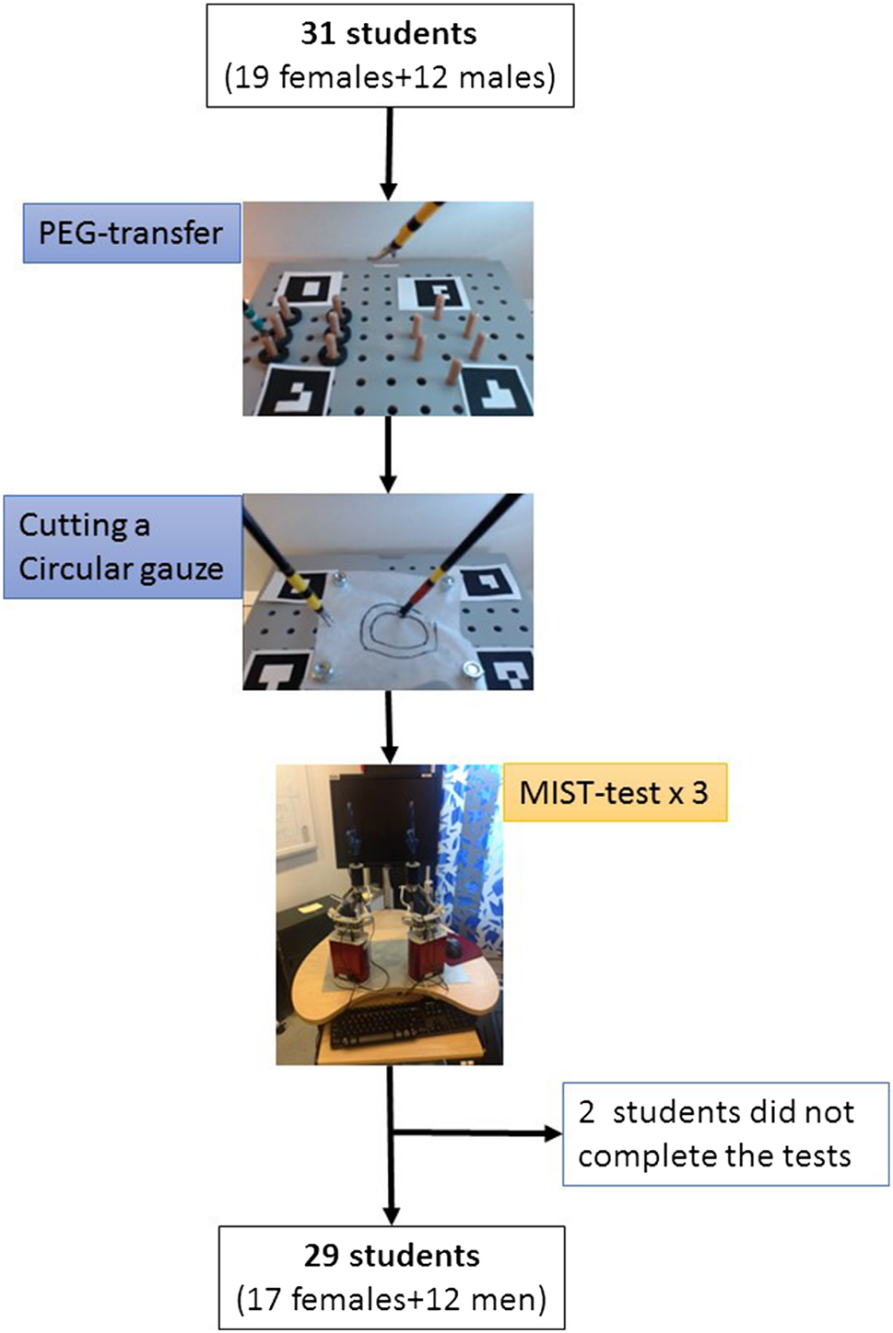
A flow sheet overview of the study including pictures of the pegboard with PEG-transfer, gauze for cutting the perimeter of a *circle*, and *markers* as reference for the video analysis software

The goal of the first task was to move a series of six rubber rings located on one side of the pegboard, to the other side of the pegboard by the use of graspers, with the non-dominant hand moving the rubber rings to the dominant one before placing them on the other side of the pegboard. This process was then reversed by switching direction and hands to bring back the rubber rings to their starting position (Fig. 2). The goal of the second task was to cut the perimeter of a 10 × 10 cm gauze without cutting the marked lines. The subjects had to cut the circular pattern avoiding both the external (diameter 5.5 cm) and internal (diameter 3.5 cm) circles. The pegboard was marked with four different markers in each corner as a reference for the video analysis software (Fig. 2) [27].

**Fig. 3:**
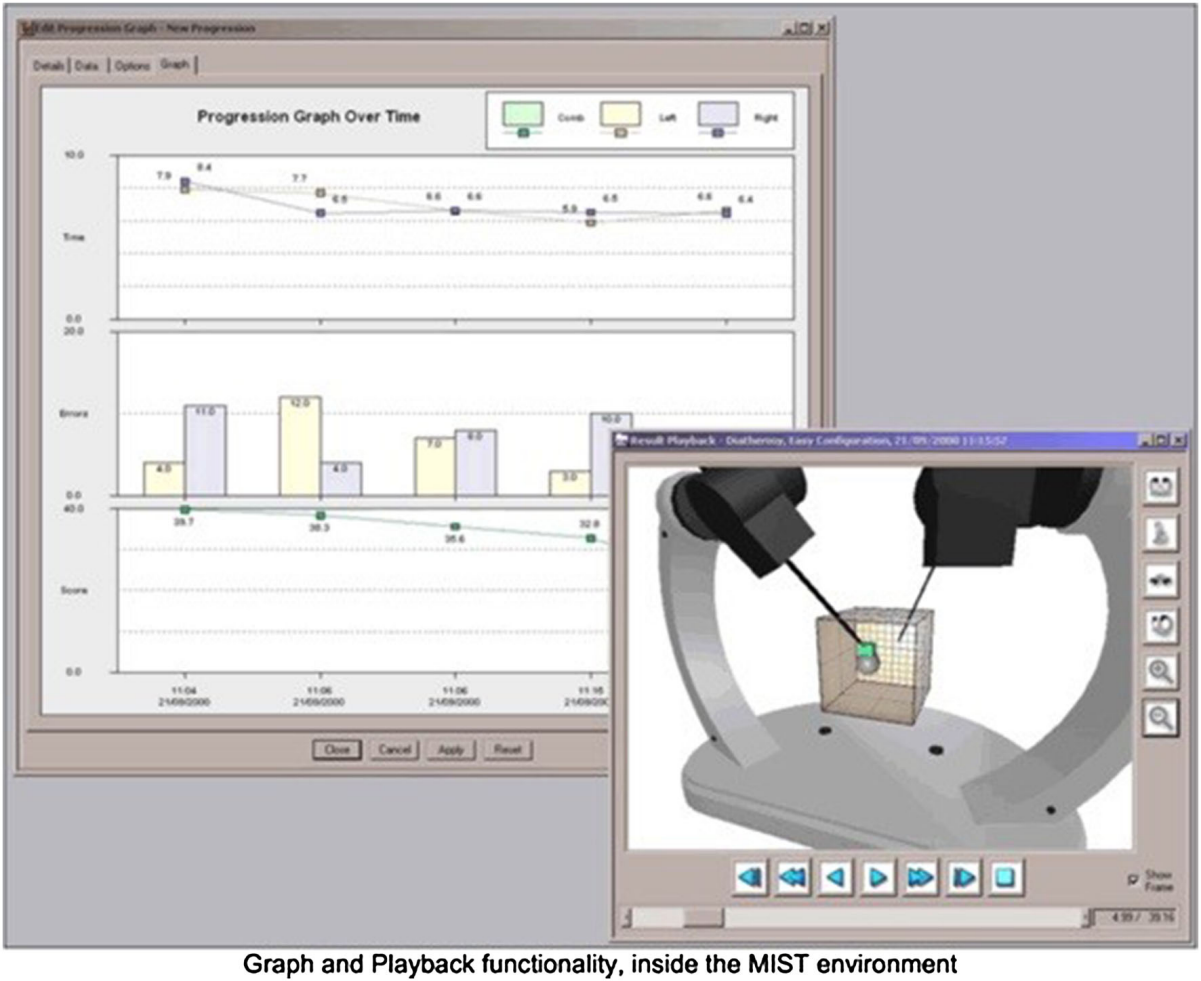
The MIST-VR score chart and handles with the ball in the 3D cube

The students then performed three consecutive manipulative diathermy medium tasks, which is a complex task on the validated MIST-VR simulator, where the test subject initially must grasp a sphere with the left handle, then touch it with the right handle, withdraw the right handle, and then once again insert it, by so transforming the right handle into a diathermy hook. The test subject had to press a foot-pedal to initiate diathermy simultaneously while placing the diathermy hook to burn a cube that appears three times on a sphere in different positions, also described by Schlickum et al. [28]. For the cube to disappear from the sphere, the subject must maintain the position at a specified 3D location inside the transparent cube with the left hand during the whole procedure (Fig. 3). After completing the procedure, the subject repeats the task with the right hand. In the MIST-VR simulator, a low score is the result of a good performance, whereas a high score indicates a poor performance. The task tests the subjects’ ability to acquire a target and apply diathermy to targets on its surface while keeping a position at a specified 3D location. Two types of graphs show the subject’s performance: *Peer to Peer*, comparing subjects within one class, and *Progression,* used to show a subject’s performance over a specified set of examinations of the same configurations (Fig. 3) [26].

### Video analysis

Each video was analyzed in a blind fashion to assess the total movement in the video based on optical flow metrics. In particular, the feature detection method [29] was applied in each video frame providing a list of image locations, and associated descriptors, suitable for tracking (hereafter referred to as ‘particles’). The algorithm used by Lucas and Kanade [30] was then used to track these particles between consecutive pairs of frames, leading to a set of motion vectors ($MV$) in each time frame, *t*:

1 $$MV_{t} = \left\{ {mv_{i} = \left( {r_{i} ,d_{i} } \right)|i = 1, \ldots ,N_{\text{p}} } \right\}_{t} ,$$

where $N_{\text{p}}$ is the number of particles detected, $r_{i} = (x_{i} ,y_{i} )$ is the position of the i*th* particle, and $d_{i} = (d_{xi} ,d_{yi} )$ is the displacement of the i*th* particle with respect to the position of the same particle detected in the previous frame. Particles with very low displacement (<2 pixels) were excluded.

Based on the aforementioned analysis, the following two metrics were derived from each video: ‘Pl’ the total displacement (‘path-length’) of all particles, and ‘Prtcl_tot’ the total number of particles detected, across all frames. No user input or training of the algorithm was required prior to the analysis. The only input was the video file, and the output was the two previous metrics.

### Data analysis

The results of the automated video analysis regarding ‘Pl’ and ‘Prtcl_tot’ were correlated to the results of the three MIST-VR scores using linear fit. The results are given as RSquare. Differences in MIST-VR scores between females and males as well as between infrequent and frequent PC gamers in the PEG-transfer exercises were analyzed using Student’s *t* test and the results given as Mean ± SEM. A *P*-value <0.05 was regarded as statistically significant. Statistical analyses were carried out using JMP^®^ version 12.1.0 (64-bit) (SAS Institute Inc).

## Results

Of the 31 participants, 29 performed all the three tasks (PEG-transfer, cutting a circular gauze, and the three MIST-VR tasks), whereas two only performed one MIST-VR task and thus were excluded (Fig. 2). There was a good linear correlation between the automated video analysis of the total path-length (Pl) in the PEG-transfer test and all three MIST-scores (RSquare 0.48, *P* < 0.0001; 0.34, *P* = 0.0009; 0.45, *P* < 0.0001) (Table 1). The total number of particles across all frame pairs of the video (Prtcl_tot) also showed a significant correlation to all three MIST-scores (Table 1). There was also a linear, although not as pronounced, correlation between Pl in the gauze cutting experiment and the three MIST-scores (RSquare 0.30, *P* = 0.0022, 0.23, *P* = 0.0082; 0.16, 0.0317, respectively), whereas Prtcl_tot only showed a significant correlation in the first two MIST-scores (Table 1).

**Table 1:** Scoring results for PEG-transfer and Cutting a circle

	PL		Prtcl_tot	
	RSquare	*P*	RSquare	*P*
PEG-transfer				
Score 1	0.48	<.0001	0.41	0.0002
Score 2	0.34	0.0009	0.29	0.0024
Score 3	0.45	<.0001	0.45	<.0001
Cutting a circle				
Score 1	0.30	0.0022	0.26	0.0044
Score 2	0.23	0.0082	0.18	0.0226
Score 3	0.16	0.0317	0.13	0.0566

**Fig. 4:**
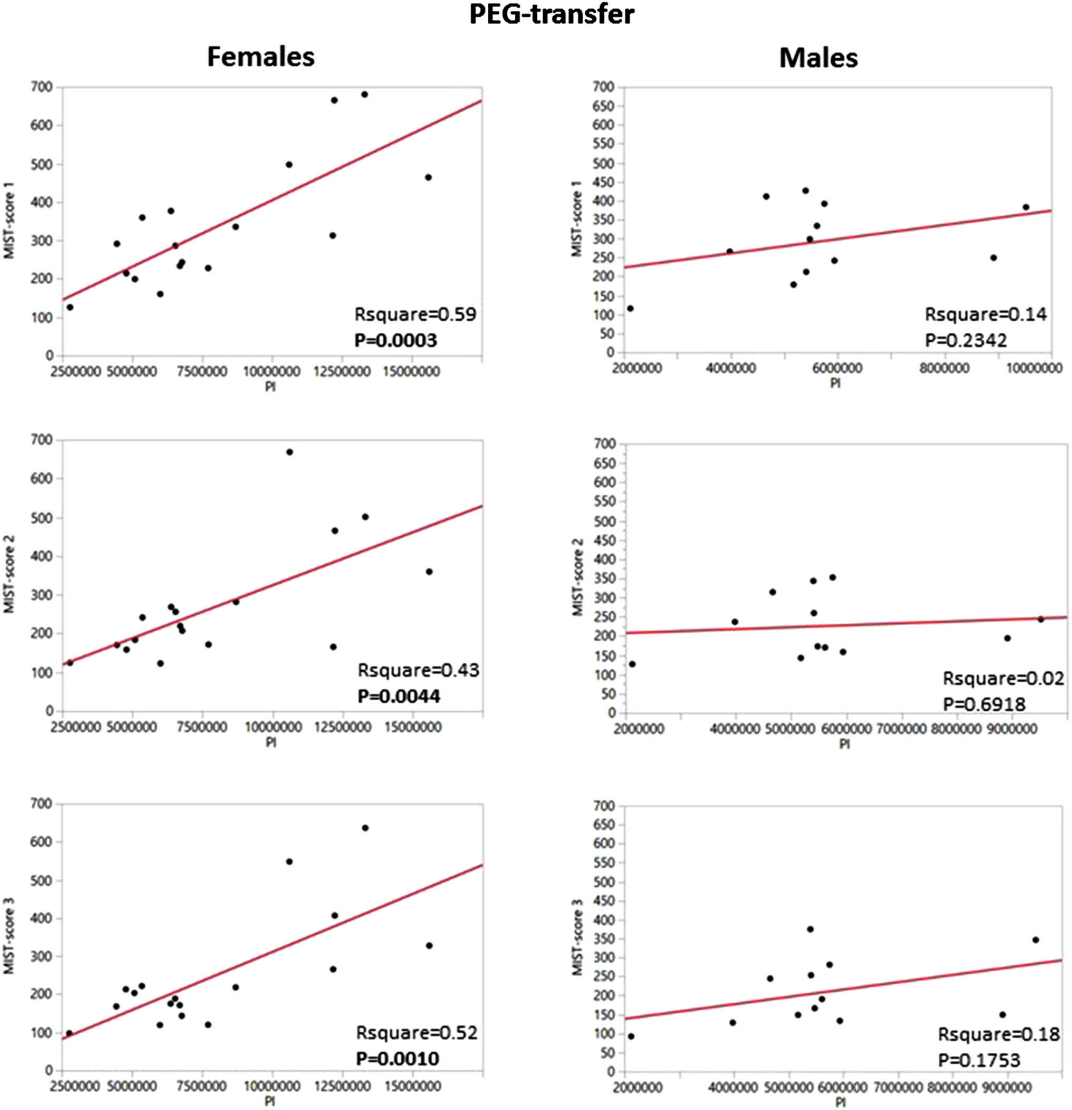
Gender-specific differences regarding correlations between the Pl variable and the MIST-VR scores in the PEG-transfer exercise

Surprisingly, there were gender-specific differences between Pl of the PEG-transfer group and the MIST-VR scores since correlations were only found in the female group (RSquare 0.59, *P* = 0.0003; 0.43, *P* = 0.0044; 0.52, *P* = 0.0010), whereas there were no correlations in the male group (Fig. 4). A similar gender-specific difference was seen in females in the gauze cutting procedure with strong linear correlations between the results of the automated video analysis and the MIST-scores (RSquare 0.51, *P* = 0.0014; 0.46, *P* = 0.0026; 0.35, *P* = 0.0127, respectively), whereas no correlations were found in the male group.

**Fig. 5:**
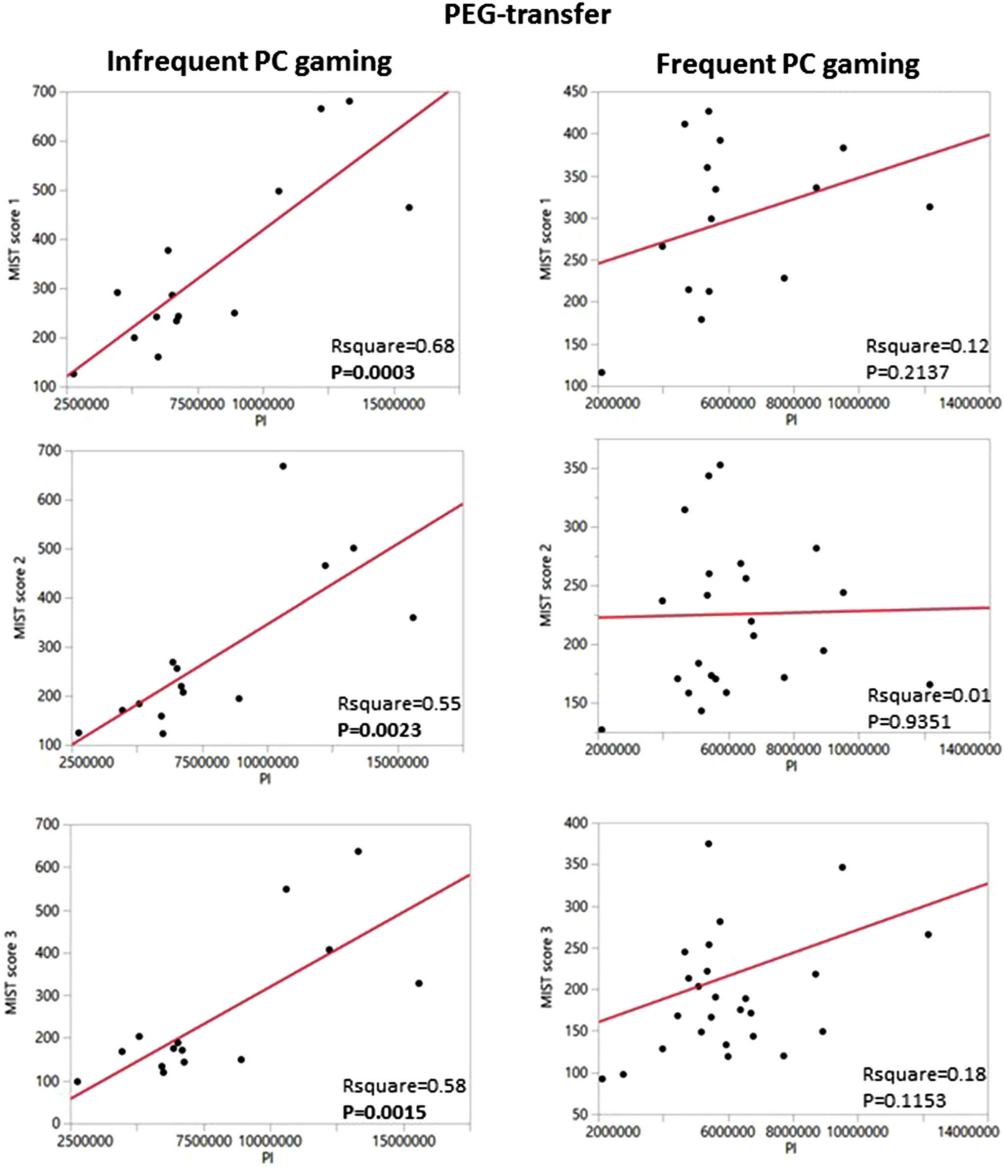
The correlation between MIST-VR score and PC gaming in the PEG-transfer exercise

Furthermore, in the PEG-transfer exercise we noted significant and even more pronounced correlations between the Pl variable in the video analysis and the MIST-VR scores (that also reflect the visuospatial haptic skills [31]) in the group with infrequent PC gaming experience, whereas in the group with the more experienced PC gamers no such correlations were found (Fig. 5). A similar pattern was also found between the Pl variable of the gauze cutting exercise in the infrequent PC gaming group and the MIST-VR 1, 2, and 3 scores, respectively (RSquare 0.49, *P* = 0.0051; 0.49, *P* = 0.0055; 0.42, *P* = 0.0118), in contrast to the frequent PC gaming group.

Although the more experienced PC gamers were predominantly males (67%), there were no significant differences in simulation performance as assessed by the MIST-simulation scores neither between males and females nor between experienced or not experienced PC gamers. We, however, noted a training effect of the simulation training since the MIST-VR scores decreased in all groups between the first and last MIST-VR simulation (low scores indicating better performance) (Table 2).

**Table 2:** MIST-VR scores in different subgroups

	Females		Males		*P*
	Mean	SEM	Mean	SEM	
Gender (PEG-transfer)					
Score 1	334	39	292	29	0.4008
Score 2	269	36	226	23	0.3345
Score 3	248	37	209	26	0.3891

	Infrequent		Frequent		*P*
	Mean	SEM	Mean	SEM	
PC gaming (PEG-transfer)					
Score 1	337	47	298	24	0.4701
Score 2	279	43	226	19	0.2776
Score 3	248	45	218	21	0.5514

## Discussion

This study shows a strong correlation between the results of the automated video analysis of the two consecutive laparoscopic tasks on a BlackBox trainer and the results of the three MIST-VR-task procedures. Furthermore, this study could also identify that the correlations between the results of the automated video analysis and the MIST-VR results were gender specific and correlated to computer gaming experience.

The findings of this study in relation to gender-specific differences regarding computer gaming experience are in accordance with other studies carried out [28, 32]. However, neither gender nor computer gaming experience significantly affected performance, when assessed by the MIST-VR scores, although there was a trend towards better performance both in males as well as in those who were experienced PC gamers. In a study by Norman et al. in 2012, the analyzed results of 24 studies comparing high-fidelity simulation (HFS) with low-fidelity simulation (LFS) showed no significant advantage of HFS [33]. In a recent study by Brinkmann et al., they actually found that when using a validated and nearly identical curriculum, the box-training group appeared to be superior in the transfer of basic skills into an experimental surgical procedure [11].

One interesting finding of the present study was that the strong correlations between automated video analysis and the performance, as assessed by the MIST-VR simulation tests, were only found in the subgroups of females and non-experienced PC gamers. These findings may indicate that the use of automated video analysis, as described in this study and elsewhere [20, 21, 34], may have its main future role in a cost-efficient screening and thereby selecting novices that would benefit more from additional basic skills training in BlackBoxes in contrast to those with more developed advanced skills that probably would benefit more from going directly to HFS-training. In surgery, an efficient use of time and resources is essential to maximize the clinical output in a safe way in the present climate of restricted working hours and limited resources. These demands should also apply to more deliberate surgical training in order to optimize expertise acquisition in surgical trainees, with the aim of improving postgraduate training programs [35].

Our finding of a training effect of the laparoscopic MIST-VR simulator exercises is in accordance with other studies [2, 3, 5, 36]. The initial [26, 36] versions of the MIST-VR lacked haptic feedback, whereas the version we used in this study had haptic feedback which is essential in simulation training. This has been also emphasized by Singapogu et al. [13]. Previous studies on the difference between BlackBoxes and VR simulators have underlined the importance of feedback in laparoscopic simulation training [37, 38]. Although the force feedback is minimal during laparoscopic procedures, the little feedback that remains is still of value. Moreover, although the MIST-VR simulator may be considered to be a bit outdated, we used it in this study since it is one of the best validated laparoscopic simulators in prospective randomized trials [4, 8].

One of the main advantages of the MIST-simulator is that it provides an instant feedback and that the exercises regarding instrument handling are similar to laparoscopic cholecystectomies although the graphics are outdated. One of the drawbacks of the automated video analysis is that the parameters measured here capture the total activity of moving objects in the video, and are not specific to the tool movement. Nevertheless, the total motion activity in the training videos is mostly due to the movement of the tools, and less to other moving objects (e.g., pegs). Moreover, in its present form [34], video analysis cannot provide instant feedback. We are aiming to shorten the time between data assessment and feedback of the analysis with the optimal aim to have the analysis online and providing instant feedback. Future studies will have to be undertaken in order to solve these problems. Another limitation of this study was that the recruitment of the participating students was on a voluntary basis and thus a selection bias of the study population cannot be excluded.

However, our model is a low-cost alternative to more advanced VR simulators that are not always available on a large scale in most countries. The financial cost of our system is in the range of US $150 and a mass production could decrease it substantially.

In conclusion, we provide evidence that in subgroups of novices there are strong correlations between our automated video analysis of BlackBox training and performance in a validated laparoscopic simulator. One potential use of our model is to assess basic skills training in a low-cost way, thus leading to enhancement of surgical performance as well as to evaluate subjects with various levels of experience.

## Footnotes

1
http://www.flsprogram.org.
